# Lymph Node Metastasis-Related Gene ITGA4 Promotes the Proliferation, Migration, and Invasion of Gastric Cancer Cells by Regulating Tumor Immune Microenvironment

**DOI:** 10.1155/2022/1315677

**Published:** 2022-10-08

**Authors:** Tianyi Fang, Xin Yin, Yufei Wang, Hao Wang, Xibo Wang, Yingwei Xue

**Affiliations:** Department of Gastroenterological Surgery, Harbin Medical University Cancer Hospital, Harbin Medical University, Harbin 150081, China

## Abstract

The Integrin Subunit Alpha 4 (ITGA4) plays important roles in cancers pathogenesis. However, the expression and association with clinicopathological and survival probability have not been previously assessed in gastric cancer (GC). Protein expression of ITGA4 was assessed in TMA using immunohistochemistry and correlated with clinicopathological factors and survival. The mRNA expression of ITGA4 was also assessed in the HMU-GC cohort. Bioinformatics function analysis was conducted through GSEA. The “CIBERSORT” package was used for immune infiltration analysis. “SvyNom” package is used to construct prognosis model. ITGA4 knock down using shRNA. The evaluation of cell function was performed by CCK-8 and Transwell invasion and migration experiments. ITGA4 was significantly associated with N classification (*P* = 0.031), tumor location (*P* = 0.033), WHO classification (*P* = 0.007), and poor prognosis in mRNA level. GSEA analysis of the validation cohort suggested that ITGA4 was associated with macrophage infiltration. Immunohistochemistry showed that ITGA4 was associated with poor prognosis. Multivariate Cox regression analysis found that ITGA4 (*P* = 0.045) and lymph node metastasis rate (*P* = 0.026) were independent prognostic factors and could construct a prognosis model. ITGA4 knockdown cell line significantly reduced the ability of proliferation, invasion, and metastasis. ITGA4 is associated with patient survival in GC and may be an important prognostic biomarker.

## 1. Introduction

Gastric cancer (GC) is one of the common malignant tumors in China. World Health Organization (WHO) reported that there were 479,000 new cases of GC and 374,000 deaths in China in 2020, accounting for 44.0% and 48.6% of the global new cases and deaths [1–3]. The incidence and mortality of GC have increased significantly, and the mortality rate of GC is the fourth among malignant tumors, which is one of the most threatening malignant tumors to the health and life of the population, resulting in a social burden that cannot be ignored [4, 5]. Lymph node metastasis plays a substantial role in the progression of GC and is one of the most indispensable factors affecting the therapeutic. The 8^th^ of the TNM staging system distinguishes patients into N3a and N3b in the final pathological stage [6]. This also shows that lymph node metastasis means irreversible tumor progression indicating that the biological characteristics of cancer cells have undergone a fundamental change and manifested as enhanced proliferation and invasion ability [7–9]. Whatever, lymph node metastasis is one of the important factors affecting the treatment and prognosis of GC. Therefore, finding biomarkers for early prediction of lymph node metastasis and developing therapeutic targets is crucial for the clinical treatment, especially for the molecular regulatory mechanisms related to lymph node metastasis, including chemotactic movement of GC cells and the formation of lymphatic vessels.

It is often found that patients with advanced GC may have significantly different prognosis even if their TNM stages are the same [10, 11]. Some GC primary sites are large in volume, but the degree of invasion is limited to the serosae, and lymph node metastasis and distant planting metastasis do not occur. If these patients are treated surgically and undergo postoperative chemotherapy according to the guidelines, the 5-year survival rate can often reach 84.0% [7]. Some patients' tumors only invade the muscle layer but have multiple lymph node metastases. Even if radical surgery, postoperative chemotherapy or preoperative neoadjuvant treatment is performed, their 5-year and 10-year survival rates are significantly reduced [12, 13]. However, studies have shown that about 60% of GC has obvious tissue heterogeneity in transcriptomics sequencing, which makes it difficult to find molecules targeted at lymph node metastasis.

Therefore, this study is different from conventional transcriptomic sequencing analysis. We started with the clinicopathological characteristics of patients and took the pathological characteristics of lymph node metastasis as a separate variable to screen out the abnormal expression of ITGA4 in tumor with positive lymph node metastasis. The full name of ITGA4 is Integrin Subunit Alpha 4. Integrin is a heterodimeric integrated membrane protein, composed of *α* Chain and *β* Chain composition, which plays a role in cell surface adhesion and signaling [14]. The precursor protein encoded by ITGA4 is proteolytically processed to produce light and heavy chains containing Alpha4 subunits. This subunit is related to *β*1 or *β*7 subunits, which binds to form integrins that may play a role in cell motility and migration [15, 16]. This integrin is a therapeutic target for the treatment of multiple sclerosis, Crohn's disease, and inflammatory bowel disease. But up to now, little research of ITGA4 in GC has been reported [17]. In this study, we try to find the possibility of ITGA4 as a biomarker and its potential mechanism in the progression of GC.

## 2. Materials and Methods

### 2.1. Overview of GC RNA Dataset and Immunohistochemical Cohort

The training cohort included 20 patients with lymph node metastatic GC with N3+ stage and 20 patients with N0 stage. The HMU-CG validation set included 246 patients underwent gastrectomy as the primary treatment in the Department of Gastroenterology, Harbin Medical University Cancer Hospital (GSE184336 and GSE179252). The quality control method is mainly completed through Agilent 2100 bioanalyzer. The kit used for library building is EBNext® Ultra™ Directional RNA Library Prep Kit for Illumina. Finally, use the Illumina platform for sequencing. All samples were collected from patients after obtaining written informed consent. The study was approved by the institutional review board of the Affiliated Tumor Hospital of Harbin Medical University. RNA isolation, library construction, and mRNA sequencing were performed by Novogene Biotech Co., Ltd. (Beijing, China). Data were stored in the Gene Expression Omnibus (GEO) repository.

Tissue microarray (TMA) samples included 180 patients underwent radical gastrectomy in the Department of Gastroenterology, Affiliated Tumor Hospital of Harbin Medical University between November 2018 and December 2019. Exclusion criteria included preoperative neoadjuvant therapy, serious heart disease, serious infectious diseases, recurrent GC, and distant metastasis.

For the preparation of tissue microarray, two experienced pathologists observed the pathological level of the whole tumor tissue through H&E staining under high-definition electron microscopy and selected a representative site to more accurately reflect the pathological characteristics of the tumor. Then, mark the position on the paraffin embedded tissue with special marker and select a sample with a diameter of 1.5 mm. The sites of sampling include tumor center and invasion front. The technical service is provided by Shanghai Aoduo Biotechnology Co., Ltd. (Shanghai, China).

### 2.2. Immunochemistry

GC tissues and paracancerous tissues were fixed in 4% paraformaldehyde. Then all tissue samples were paraffin embedded and prepared for at least 4 *μ*m thick tissue sections. Pathologists performed hematoxylin and eosin (H&E) staining to select the representative location for preparing tissue chips, and the lattice diameter was 15 mm. In order to detect the protein expression in the tissue, the sections were first repaired with EDTA antigen repair solution in a 120°C pressure cooker environment for 3 minutes, then incubated with 3% H_2_O_2_ at room temperature for 30 minutes, goat serum at room temperature for 2 hours, and then incubated with primary antibodies specific for ITGA4 at 4°C overnight (1 : 1000, Affinity, #DF6135). The secondary antibodies (Elabscience, No.E-IR-R217) were incubated at room temperature for 30 minutes, and the chromogenic reaction was performed via diaminobenzidine (DAB) staining. The assessment of immunohistochemistry used the H-score method. This score was derived from the dyeing intensity and was scored as negative (0), weak (1), medium (2), or strong (3) multiplied by percentage of dyed area under intensity. Immunohistochemical score data were divided into high expression or low expression according to survival rate by X-tile software.

### 2.3. Cell Culture

All cell lines (GES, AGS, BGC-823, HGC-27, MKN-28, and 293T) were obtained from the cell line service (Procell Biotechnology Co., Ltd., Wuhan, China) and authenticated by the provider. These cell lines were not included in the misidentified cell line database of the The International Cell Line Authentication Committee (ICLAC) (http://iclac.org/). In this study, BGC-823 cell line was used to investigate the effect of ITGA4 on the proliferation, migration, and invasion of GC. 293T cells were used for lentiviral infection. GES, BGC-823, HGC-27, and MKN-28 cell lines were cultured using modified McCoy's 5A medium RPMI-1640 (PM150110), 10% fetal bovine serum (164210-50), 1% Penicillin-Streptomycin Solution (PB180120), and 1% GlutaMax (PB180419). The AGS cell line used Ham's F-12 nutrient mixture (PM150810), 10% fetal bovine serum (164210-50), and 1% Penicillin-Streptomycin Solution (PB180120). The 293T cell line was cultured in Dulbecco's modified medium DMEM (PM150210), 10% fetal bovine serum (164210-50), and 1% Penicillin-Streptomycin Solution (PB180120). All cell lines were cultured in a humidified incubator containing 5% CO_2_ at 37°C.

### 2.4. Cell Transfection

The shRNA against ITGA4 was designed and synthesized according to the known sequence, and then inserted into the lentiviral expression vector GV493 (Genechem, China). The sequences of shRNA targeting IGTA4 are shown in Supplementary Table S1. This lentivirus vector was transfected into 293T cells together with the packaging system plasmids psPAX2 and pMD2.G to obtain pseudolentivirus particles. 20 *μ*g lentiviral vector, 15 *μ*g psPAX2, and 10 *μ*g pMD2.G were used to transfect a 5 × 10^6^ 293T cells. The supernatant of 293T was collected and concentrated by an ultracentrifuge at 25 000 rpm for 2 h at 4°C. BGC-823 cells were transduced with concentrated lentiviral particles. IGTA4 knock down was verified by Western blot. Scrambled sequences were inserted into these vectors for use as controls.

### 2.5. Western Blot

The proteins were extracted from gastric epithelial cells and cancer cells, and the extracted proteins were quantified using BCA Kit (Beyotime Institute of Biotechnology). The proteins were separated by sodium dodecyl sulphate-polyacrylamide gel electrophoresis (SDS-PAGE), transferred to PVDF membrane (Merck Millipore Ltd.), and sealed with BSA (5%) under RT for 2 hours. Subsequently, the membrane was incubated with ITGA4 specific primary antibody at 4°C overnight (1 : 1000, Affinity, DF6135). The membranes were incubated with horseradish peroxidase labeled secondary antibody at 37°C for 40 minutes, and then visualize by ECL (Thermo Scientific).

### 2.6. Cell Counting Kit-8 (CCK-8) Assay

Cell viability in vitro was evaluated by cell counting kit-8 (CCK-8) assay. 4 × 10^3^ cells were implanted into 96-well plates. After the cells adhered to the wall after 4 hours of culture, the medium in each well was replaced with 100 ml RPMI-1640 serum-free medium, and the adsorption rate after 2 hours was measured by a microplate reader at 450 nm in a medium containing 10 ml CCK-8 reagent (Meilunbio, MA0218-3). Then repeat the detection at 24 h, 48 h, and 72 h, respectively.

### 2.7. Cell Migration and Invasion Assay

Migration and invasion analyses were performed using a 24-well Transwell chamber system (Costar, USA, #3422). Briefly, cells were resuspended in serum-free medium, washed twice, and seeded into the upper chamber (8 × 10^4^ cells per 200 *μ*l). The lower chamber contains 900 *μ*l medium containing 20% FBS. After 24 hours of incubation, the cells were fixed with 95% ethanol for 15 minutes, and then stained with 0.1% crystal violet for 30 minutes. Use a cotton swab to remove the cells that failed to penetrate the filter from the upper chamber. Under the light microscope, migrating and invading cells were counted in three randomly selected regions. For the invasion test, the chamber was coated with 40 *μ*l Corning Matrix Matrix (#356234, USA) before cell inoculation.

### 2.8. Statistical Analysis and Bioinformatics Analysis

Data were analyzed using SPSS 22.0 (Chicago, USA, SPSS Inc.) software and shown as mean ± SD. Chi-square test was used to evaluate the relationship between ITGA4 expression and clinicopathological features if they meet the conditions that the theoretical frequency is more than 5 and the total sample size is more than 40. If they do not meet the conditions that the theoretical frequency is more than 5 or the total sample size is more than 40, so the Fisher exact test is recommended. The Wilcoxon rank sum test is selected if the normal distribution is not satisfied. Survival analysis of patients was analyzed using log-rank test and Cox regression. Survival curves and overall survival (OS) were determined by Kaplan-Meier and log Rank methods. Gene Ontology (GO) pathway enrichment analysis was used for genome functional annotation. The “clusterprofiler” package was used to study the functional enrichment of risk score-related genes in gene set enrichment analysis (GSEA). All bioinformatics analyses were performed using R Studio software (v4.0.2). A two tailed *P* value < 0.05 is considered significant.

## 3. Results

### 3.1. ITGA4 Was Highly Correlated with Lymph Node Metastasis in the Training Cohort

**Figure 1:**
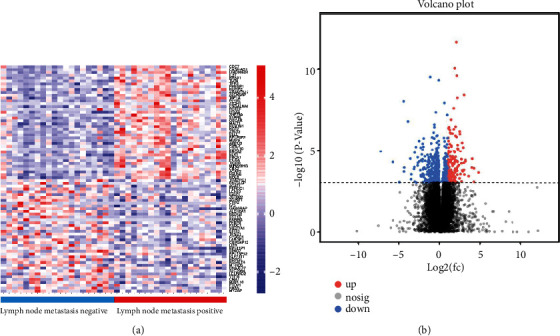
(a) Heatmap showing genes associated with lymph node metastasis of GC. (b) Volcano plot shows the differentially expressed genes between the two groups of samples.

In this study, we first searched for ITGA4 related to lymph node metastasis through transcriptome-level sequencing. There were 20 patients in each of the two groups in the training cohort. The pathological data were not statistically significant except for lymph node metastasis (Supplementary Table S2). The sequencing results showed that ITGA4 gene was significantly overexpressed in patients with positive lymph node metastasis (*P* < 0.001, Figure 1(a)).  Figure 1(b) shows the differential genes of the two groups. In the group with positive lymph node metastasis, 309 genes were upregulated and 331 genes were downregulated.

### 3.2. ITGA4 Expression Was Associated with Poor Clinicopathological Features in the Validation Cohort

**Figure 2:**
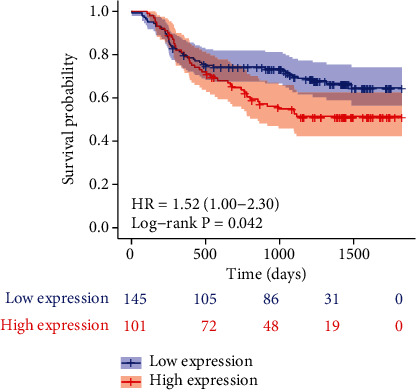
Kaplan–Meier survival analysis of the OS for patients with different ITGA4 expression in HMU-GC validation cohort.

We examined the relationship between ITGA4 mRNA expression and OS, clinicopathological features in the HMU-GC validation cohort. The cut-off of mRNA was calculated according to the ROC curve. There were 101 patients with high expression of ITGA4 and 145 patients with low expression. Kaplan-Meier analysis showed that ITGA4 was associated with poor prognosis, and the prognosis of patients with high expression of ITGA4 had a lower survival probability (Figure 2, *P* = 0.045). In addition, ITGA4 was highly correlated with poor pathological features such as N classification (*P* = 0.031), tumor location (*P* = 0.033), and WHO classification (*P* = 0.007) (Table 1).

### 3.3. Bioinformatics Analysis of the Validation Cohort Suggested that ITGA4 Was Associated with Macrophage Infiltration

**Figure 3:**
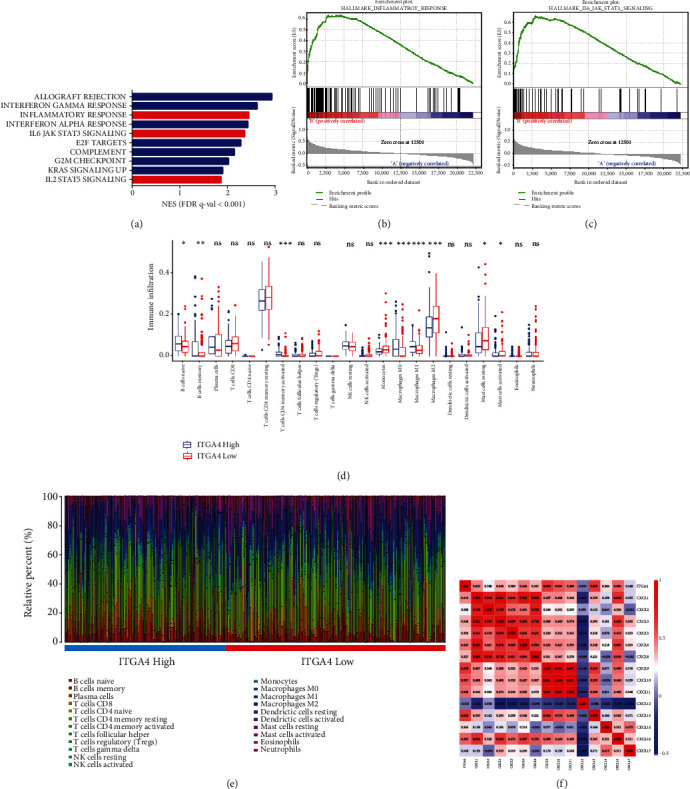
(a) ITGA4 expression correlated with inflammatory response, (b) IL6 JAK STAT3 signaling, (c) and IL2 STAT5 signaling from the HMU-GC validation cohort. (d, e) Analysis of immune cell components according to different ELN expression levels from the HMU-GC validation cohort. (f) Correlation analysis of ITGA4 and chemokines.

We performed GSEA analysis related to ITGA4 in the HMU-GC validation cohort (Figure 3(a)), and the results showed that immune response pathways such as inflammatory response (Figure 3(b)) and IL6 JAK STAT3 signaling (Figure 3(c)) were activated in the tissues of patients with high expression of ITGA4 (*P* < 0.001). In order to explore which kind of immune cells plays the more important role in GC tissues with different levels of ITGA4, we analyzed the immune cell composition with different levels of expression of ITGA4. The results showed that macrophages were enriched higher in tissues with high expression of ITGA4 (Figure 3(d) and 3(e)). At the same time, we also found that ITGA4 maintained this immune response mainly through a variety of chemokines, especially CXCL9 (*r* = 0.630) and CXCL1 (*r* = 0.628, Figure 3(f)).

### 3.4. Immunohistochemistry Showed that ITGA4 Was Associated with Poor Prognosis and Could Construct a Prognosis Model

**Figure 4:**
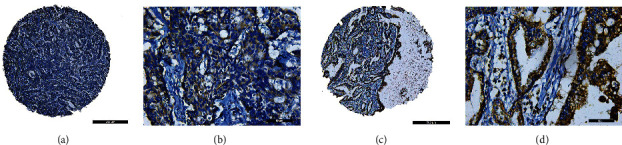
ITGA4 immunohistochemistry staining of TMA. (a) Low expression of ITGA4 at ×50. (b) Low expression of ITGA4 at ×400. (c) High expression of ITGA4 at ×50. (d) High expression of ITGA4 at ×400.

**Figure 5:**
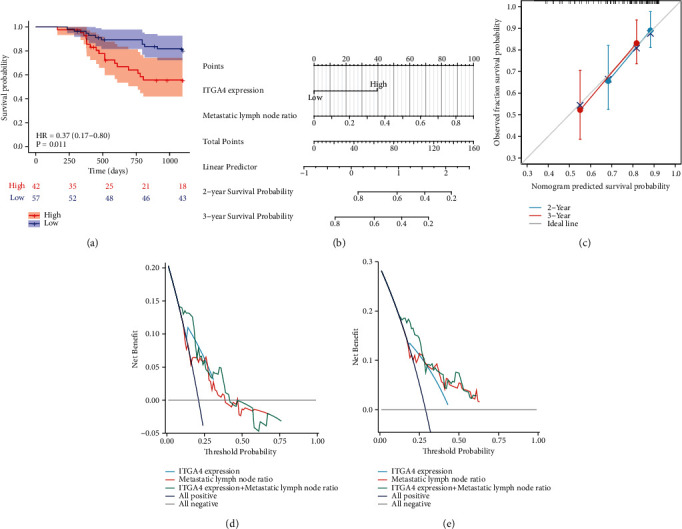
(a) Kaplan–Meier survival analysis of the OS for patients with different ITGA4 expression levels in TMAs. (b) Nomogram prognostic model. (c) Calibration analysis in 2 and 3 years. (d) Decision curve analysis in 2 years. (e) Decision curve analysis in 3 years.

We evaluated the expression of ITGA4 through immunohistochemistry, which is mainly expressed in the cytoplasm of tumor cells (Figure 4). Chi-square analysis showed that ITGA4 was mainly related to T classification (*P* = 0.028), N classification (*P* = 0.006), lymph node metastasis rate (*P* = 0.018), and serum CA19-9 (*P* = 0.003) (Table 2), which was basically consistent with the results of previous transcriptome level analysis. There are 180 samples of TMA, and 100 samples meet the 3-year follow-up, so the survival analysis includes 100 samples. Survival analysis showed that high expression of ITGA4 was significantly related to poor patient survival (Figure 5(a), *P* = 0.011). Univariate Cox regression analysis showed that ITGA4 (*P* = 0.011), lymph node metastasis rate (*P* < 0.001), and tumor location (*P* = 0.003) were correlated with prognosis. Multivariate Cox regression analysis found that ITGA4 (*P* = 0.045) and lymph node metastasis rate (*P* = 0.026) were independent prognostic factors (Table 3). Next, we construct a prognosis model (Figure 5(b)) based on independent prognostic factors of Cox regression analysis. The calibration curve analysis of the prognosis model has satisfactory accuracy, C − index = 0.705 (0.656-0.753) (Figure 5(c)). DCA analysis showed that the prognosis model had an acceptable predictive value in predicting the death risk in 2 years (Figure 5(d)) and 3 years (Figure 5(e)), ITGA4 C − index = 0.623 (0.577-0.669), the lymph node metastasis rate C − index = 0.647 (0.593-0.702), and the combined C − index = 0.705 (0.656-0.753).

### 3.5. ITGA4 Knockdown Cell Line Significantly Reduced the Ability of Proliferation, Invasion, and Metastasis

**Figure 6:**
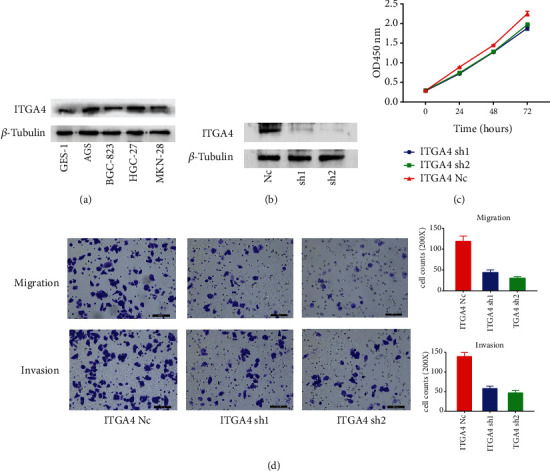
(a) Protein expression of ITGA4 in gastric epithelium and different GCs. (b) Verification of ITGA4 knockdown in protein level. (c) CCK-8 analysis of ITGA4 knockdown in BGC-823 cell line. (d) Transwell migration and invasion assay in BGC-823 cell line after ITGA4 knockdown.

We first verified at the protein level that ITGA4 is generally highly expressed in GC cells compared with gastric epithelial cells (Figure 6(a)). Then we constructed the ITGA4 knockdown cell line of BGC-823 and verified it at the protein level (Figure 6(b)). Next, we evaluated the ability of ITGA4 to promote GC proliferation through CCK-8 experiment. The results showed that the proliferation ability of BGC-823 cell lines with ITGA4 knockdown was significantly inhibited than that of the control group (Figure 6(c)). The results of previous clinical immunohistochemical experiments suggested that the expression of ITGA4 was significantly correlated with the metastasis rate of lymph nodes. We tested the effect of ITGA4 gene on the migration and invasion ability of GC cells through Transwell experiments. The results showed that after knockdown of ITGA4 by BGC-823 cell line, the number of cells penetrating the membrane in migration and invasion simulation experiment was significantly less than that in the control group (Figure 6(d)).

## 4. Discussion

The TNM staging system based on tumor invasion, regional lymph node, and distant metastasis in GC is recognized as an international standard to predict prognosis and guide postoperative treatment [18]. Adjuvant chemotherapy is recommended for patients with stage II or III pathological stage after radical surgery to reduce recurrence [5]. However, how to more accurately evaluate lymph node metastasis is a clinical problem that surgeons and pathologists have been working on for a long time. Therefore, we want to screen a reliable biomarker according to the difference of lymph node metastasis to evaluate the biological function and prognosis of GC. In this study, we first found that ITGA4 was highly correlated with lymph node metastasis in GC. Then, we analyzed the mRNA and immunohistochemical expression of ITGA4 and the relationship between expression level and clinicopathological factors. We found that the high expression of ITGA4 affects the immune infiltration status of cancer tissues, especially related to the enrichment of macrophages. We further confirmed that ITGA4 can promote GC cell migration and invasion in vitro. Finally, we constructed a prognostic model for GC based on ITGA4 expression and clinicopathological characteristics.

ITGA4 (Integrin Subunit Alpha 4) is a protein-coding gene. The gene encodes a member of the integrin alpha chain family of proteins. Integrin is a member of the superfamily of transmembrane glycoproteins *α* and *β* and a heterodimer membrane receptor protein composed of two subunits that function in cell surface adhesion and signaling. ITGA4 has been proved to be involved in cell proliferation, apoptosis, adhesion, and migration, which promoting tumor progression [19]. In addition, methylation of ITGA4 exists in many kinds of primary tumors, which may play a key role in the transformation of inflammatory cancer [20–22]. However, there are little studies that have confirmed the prognostic value of ITGA4 in GC. In this study, we found that high expression of ITGA4 was significantly associated with vascular invasion and lymph node metastasis. At the same time, we further confirmed that ITGA4 promotes GC cell migration and invasion, which suggests that it may be a potential prognostic biomarker. Survival analysis showed that ITGA4 had satisfactory prognostic value in GC patients.

In order to further verify the reliability of ITGA4 as a biomarker, we found that ITGA4 is significantly related to the activation of IL6/JAK/STAT3, IL2/STAT5, and other immune response pathways through GSEA and GO enrichment analysis. In addition, the analysis of immune cell components in cancer tissues showed that there was more macrophages that were enriched in the tissues with high expression of ITGA4. Wei et al. [23] reasonably explains this result that tumor associated macrophages (TAMs-) derived IL-6 activated the JAK2/STAT3 signaling pathway. The activated STAT3 transcriptional inhibition produces CCL2 to promote macrophage recruitment. It is worth noting that our results show that the overexpression of ITGA4 was related to the enrichment of M1 type TAMs. This is contrary to previous cognition. Previous studies have shown that TAMs can be polarized into tumor supporting M2 like macrophages and tumor inhibiting M1 like macrophages [24–26]. You et al. [27] found that M1 like TAMs significantly promoted the epithelial mesenchymal transformation (EMT) process and induced the formation of cancer stem cells by upregulating the expression of MME and MMP14 in oral squamous cell carcinoma. M1 like TAMs promote the invasion and migration of cell colonies by activating the JAK/STAT3 pathway. This is consistent with our results, indicating that M1 like TAMs do not play a diversified inhibitory role in tumor progression. In addition, our results show that undifferentiated M0 type TAMs is enriched in tissues with high expression of ITGA4. This means that the high expression of ITGA4 is closely related to macrophage polarization and may induce more TAMs [28]. In the future, researches should pay more attention to how ITGA4 in the GC microenvironment affects the dynamic polarization of macrophages. At the same time, our research results confirm that ITGA4 mainly maintains this specific immune infiltration state through a variety of chemokines, such as CXCL9. Relevant studies have found that M1 like TAMs may recruit CD8^+^ tissue resident memory (TRM) T cells through CXCL9 overexpression and provide TRM with essential fatty acids to maintain immune infiltration [29].

Of course, our research has some limitations. First, the analysis of mRNA markers and immunoassays in tumors comes from transcriptome level sequencing, which has not been verified by rigorous molecular level experiments, and should be interpreted carefully. Second, functional characterization and verification of the role of ITGA4 in immune cells will be very important to determine whether the cancer promoting effect of ITGA4 in the GC microenvironment is a driver or a bystander. In the survival analysis of ITGA4, it is also necessary to conduct multicenter and multiethnic trials in a larger cohort to verify the role of ITGA4 in the treatment response to patient survival and other clinical features, such as immune efficacy and metastasis status.

## Figures and Tables

**Table 1:** Relationship between ITGA4 mRNA expression and the clinical characteristics of GC in the HMU-GC validation cohort.

Characteristic	High expression (*n* = 101)	Low expression (*n* = 145)	*P* value
Sex, *n* (%)			0.711
Female	38 (15.4%)	50 (20.3%)	
Male	63 (25.6%)	95 (38.6%)	
T classification, *n* (%)			0.241
T1a	2 (0.8%)	4 (1.6%)	
T1b	1 (0.4%)	6 (2.4%)	
T2	7 (2.8%)	10 (4.1%)	
T3	64 (26%)	101 (41.1%)	
T4a	14 (5.7%)	16 (6.5%)	
T4b	13 (5.3%)	8 (3.3%)	
N classification, *n* (%)			0.031
N0	17 (6.9%)	32 (13%)	
N1	10 (4.1%)	19 (7.7%)	
N2	17 (6.9%)	25 (10.2%)	
N3a	28 (11.4%)	51 (20.7%)	
N3b	29 (11.8%)	18 (7.3%)	
M classification, *n* (%)			0.806
M0	95 (38.6%)	134 (54.5%)	
M1	6 (2.4%)	11 (4.5%)	
pTNM, *n* (%)			0.077
IA	3 (1.2%)	8 (3.3%)	
IB	4 (1.6%)	6 (2.4%)	
IIA	10 (4.1%)	13 (5.3%)	
IIB	7 (2.8%)	16 (6.5%)	
IIIA	18 (7.3%)	28 (11.4%)	
IIIB	24 (9.8%)	46 (18.7%)	
IIIC	29 (11.8%)	17 (6.9%)	
IV	6 (2.4%)	11 (4.5%)	
Borrmann type, *n* (%)			0.307
Borrmann I	4 (1.6%)	1 (0.4%)	
Borrmann II	22 (8.9%)	28 (11.4%)	
Borrmann III	60 (24.4%)	89 (36.2%)	
Borrmann IV	15 (6.1%)	27 (11%)	
Tumor location, *n* (%)			0.033
Lower third	41 (16.7%)	77 (31.3%)	
Middle third	20 (8.1%)	35 (14.2%)	
Upper third	22 (8.9%)	21 (8.5%)	
Entire stomach	18 (7.3%)	12 (4.9%)	
Lymphatic infiltration, *n* (%)			0.604
Negative	46 (18.7%)	60 (24.4%)	
Positive	55 (22.4%)	85 (34.6%)	
Nerve infiltration, *n* (%)			0.677
Negative	24 (9.8%)	30 (12.2%)	
Positive	77 (31.3%)	115 (46.7%)	
WHO classification, *n* (%)			0.007
Mucinous	8 (3.3%)	1 (0.4%)	
Poorly differentiated	26 (10.6%)	38 (15.4%)	
Signet ring cell	39 (15.9%)	47 (19.1%)	
Well to moderately differentiated	28 (11.4%)	59 (24%)	
Age, median (IQR)	61 (51, 67)	58 (47, 64)	0.144
CEA, median (IQR)	1.81 (1.16, 3.85)	2.25 (1.27, 3.9)	0.245
CA-199, median (IQR)	10.91 (6.17, 22.47)	11.57 (5.16, 22.81)	0.714
CA724, median (IQR)	2.49 (1.29, 11.86)	3.03 (1.29, 6.64)	0.706
CA125, median (IQR)	10.21 (8.08, 13.47)	10.21 (7.72, 15.67)	0.797

Histological type, T classification, N classification, and pTNM classification were according to the AJCC 8th edition of the Cancer Staging Manual of the American Joint Committee on Cancer. Vascular infiltration, nerve infiltration, and lymphatic infiltration were determined according to the postoperative pathology report. IQR: interquartile range.

**Table 2:** The relationship between ITGA4 expression in tumor tissue and the clinicopathological characteristics.

Characteristic	High expression (*n* = 74)	Low expression (*n* = 106)	*P* value
Age, *mean* ± *SD*	58.93 ± 9.75	61.2 ± 9.16	0.114
BMI, median (IQR)	22.51 (20.21, 24.48)	23.19 (21.12, 25.45)	0.092
Sex, *n* (%)			0.402
Female	16 (8.9%)	30 (16.7%)	
Male	58 (32.2%)	76 (42.2%)	
Tumor infiltration pattern, *n* (%)			0.189
INFa	12 (6.7%)	24 (13.3%)	
INFb	14 (7.8%)	30 (16.7%)	
INFc	34 (18.9%)	34 (18.9%)	
N/A	14 (7.8%)	18 (10%)	
Lymphatic infiltration, *n* (%)			0.163
Negative	47 (26.1%)	55 (30.6%)	
Positive	27 (15%)	51 (28.3%)	
Venous infiltration, *n* (%)			0.936
Negative	55 (30.6%)	77 (42.8%)	
Positive	19 (10.6%)	29 (16.1%)	
Nerve infiltration, *n* (%)			0.530
Negative	17 (9.4%)	30 (16.7%)	
Positive	57 (31.7%)	76 (42.2%)	
T classification, *n* (%)			0.028
T1	5 (2.8%)	5 (2.8%)	
T2	6 (3.3%)	21 (11.7%)	
T3	24 (13.3%)	44 (24.4%)	
T4	39 (21.7%)	36 (20%)	
N classification, *n* (%)			0.006
N0	18 (10%)	32 (17.8%)	
N1	8 (4.4%)	28 (15.6%)	
N2	17 (9.4%)	24 (13.3%)	
N3	31 (17.2%)	22 (12.2%)	
pTNM classification, *n* (%)			0.109
I	9 (5%)	14 (7.8%)	
II	17 (9.4%)	39 (21.7%)	
III	48 (26.7%)	53 (29.4%)	
Metastatic lymph node ratio, *n* (%)			0.018
< 0.3	50 (27.8%)	90 (50%)	
≥ 0.6	8 (4.4%)	4 (2.2%)	
0.3 ≥, < 0.6	16 (8.9%)	12 (6.7%)	
Borrmann type, *n* (%)			0.330
Borrmann I	6 (3.3%)	9 (5%)	
Borrmann II	20 (11.1%)	29 (16.1%)	
Borrmann III	38 (21.1%)	62 (34.4%)	
Borrmann IV	10 (5.6%)	6 (3.3%)	
Lauren classification, *n* (%)			0.514
Diffuse	30 (16.7%)	34 (18.9%)	
Intestinal	17 (9.4%)	32 (17.8%)	
Mixed	13 (7.2%)	23 (12.8%)	
Unknown	14 (7.8%)	17 (9.4%)	
Family cancer history, *n* (%)			0.441
No	69 (38.3%)	94 (52.2%)	
Yes	5 (2.8%)	12 (6.7%)	
Tumor location, *n* (%)			0.058
Entire stomach	5 (2.8%)	1 (0.6%)	
Lower third	35 (19.4%)	62 (34.4%)	
Middle and upper third	34 (18.9%)	43 (23.9%)	
HER2 expression, *n* (%)			0.330
Negative	61 (33.9%)	94 (52.2%)	
Positive	13 (7.2%)	12 (6.7%)	
CEA, *n* (%)			0.354
> 5 ng/ml	12 (6.7%)	11 (6.1%)	
≤ 5 ng/ml	62 (34.4%)	95 (52.8%)	
CA19-9, *n* (%)			0.003
> 37 U/ml	16 (8.9%)	6 (3.3%)	
≤ 37 U/ml	58 (32.2%)	100 (55.6%)	
CA72-4, n (%)			0.530
> 6 U/ml	17 (9.4%)	30 (16.7%)	
≤ 6 U/ml	57 (31.7%)	76 (42.2%)	

Histological type, T classification, N classification, and pTNM classification were according to the AJCC 8th edition of the Cancer Staging Manual of the American Joint Committee on Cancer. Vascular infiltration, nerve infiltration, and lymphatic infiltration were determined according to the postoperative pathology report. IQR: interquartile range; SD: standard deviation.

**Table 3:** Univariate and multivariate Cox analysis of ITGA4 expression and the clinicopathological variables.

Characteristics	Total (*N*)	Univariate analysis		Multivariate analysis	
		Hazard ratio (95% CI)	*P* value	Hazard ratio (95% CI)	*P* value
Group	100				
High expression	43	Reference			
Low expression	57	0.374 (0.174-0.801)	0.011	0.450 (0.206-0.984)	0.045
Sex	100				
Female	28	Reference			
Male	72	1.175 (0.499-2.765)	0.712		
Age	100	0.992 (0.957-1.028)	0.646		
BMI	100	0.944 (0.845-1.054)	0.303		
Tumor infiltration pattern	100				
INFc	48	Reference			
INFb	16	0.843 (0.280-2.541)	0.761		
INFa	20	0.568 (0.188-1.712)	0.315		
N/A	16	1.035 (0.376-2.848)	0.947		
Lymphatic infiltration	100				
Positive	45	Reference			
Negative	55	1.064 (0.503-2.249)	0.872		
Venous infiltration	100				
Positive	30	Reference			
Negative	70	1.689 (0.685-4.166)	0.255		
Nerve infiltration	100				
Positive	75	Reference			
Negative	25	0.446 (0.155-1.286)	0.135		
T classification	100				
T4	38	Reference			
T3	45	0.591 (0.268-1.302)	0.192		
T2	13	0.348 (0.079-1.533)	0.163		
T1	4	0.585 (0.077-4.452)	0.605		
Metastatic lymph node ratio	100	14.056 (3.348-59.004)	<0.001	7.032 (1.262-39.187)	0.026
Borrmann type	100	1.445 (0.769-2.715)	0.253		
Tumor location	100				
Lower third	54	Reference			
Middle and upper third	42	1.866 (0.847-4.113)	0.122	1.369 (0.590-3.177)	0.464
Entire stomach	4	7.426 (2.017-27.337)	0.003	1.838 (0.376-8.988)	0.452
HER2	100				
Positive	18	Reference			
Negative	82	0.602 (0.256-1.418)	0.246		
CEA	100				
≤ 5 ng/ml	86	Reference			
> 5 ng/ml	14	0.679 (0.205-2.250)	0.526		
CA-199	100				
≤ 37 U/ml	88	Reference			
> 37 U/ml	12	1.745 (0.663-4.593)	0.260		
CA724	100				
≤ 6 U/ml	74	Reference			
> 6 U/ml	26	1.096 (0.483-2.490)	0.826		

Histological type and T classification were according to the AJCC 8th edition of the Cancer Staging Manual of the American Joint Committee on Cancer. Vascular infiltration, nerve infiltration, and lymphatic infiltration were determined according to the postoperative pathology report.

## Data Availability

The datasets used in this study are available from the corresponding author on reasonable request. More information can also be obtained from the Gastric Cancer Information Management System v1.2 of Harbin Medical University Cancer Hospital (Copyright No. 2013SR087424, http://www.sgihmu.com/). The datasets HMU-training cohort and HMU-validation cohorts presented in this study can be found in online repositories (GSE184336 and GSE179252).

## Supplementary Materials

Supplementary Materials Table S1: the shRNA designed for ITGA4 knockdown. Table S2: baseline characteristics in the training cohort.
