# Supplementary material for: Lymph Node Metastasis-Related Gene ITGA4 Promotes the Proliferation, Migration, and Invasion of Gastric Cancer Cells by Regulating Tumor Immune Microenvironment
Source: J Oncol. 2022 Oct 8;2022:1315677. doi: 10.1155/2022/1315677 (PMC9569201; doi:10.1155/2022/1315677)
Supplement: Supplementary Materials — Table S1: the shRNA designed for ITGA4 knockdown. Table S2: baseline characteristics in the training cohort. [file 1315677.f1.docx]

Table S1. The shRNA designed for ITGA4 knockdown.

| **ID** | **5’** | **stem** | **loop** | **stem** | **3’** |
| --- | --- | --- | --- | --- | --- |
| ITGA4-RNAi(114711-1)-a | Ccgg | TGTGGAAAGACTTGTTTGGAA | CTCGAG | TTCCAAACAAGTCTTTCCACA | TTTTTg |
| ITGA4-RNAi(114711-1)-b | aattcaaaaa | TGTGGAAAGACTTGTTTGGAA | CTCGAG | TTCCAAACAAGTCTTTCCACA |  |
| ITGA4-RNAi(114713-1)-a | Ccgg | AGATGCAGGATCGGAAAGAAT | CTCGAG | ATTCTTTCCGATCCTGCATCT | TTTTTg |
| ITGA4-RNAi(114713-1)-b | aattcaaaaa | AGATGCAGGATCGGAAAGAAT | CTCGAG | ATTCTTTCCGATCCTGCATCT |  |

Table S2. Baseline characteristics in the training cohort.

| Characteristic | Group N3+ (n=20) | Group N0 (n=20) | *P* Value |
| --- | --- | --- | --- |
| Sex, n (%) |  |  | 0.731 |
| female | 7 (17.5%) | 5 (12.5%) |  |
| male | 13 (32.5%) | 15 (37.5%) |  |
| T classification, n (%) |  |  | 0.276 |
| T3 | 16 (40%) | 13 (32.5%) |  |
| T4a | 1 (2.5%) | 5 (12.5%) |  |
| T4b | 3 (7.5%) | 2 (5%) |  |
| N classification, n (%) |  |  | **< 0.001** |
| N0 | 20 (50%) | 0 (0%) |  |
| N3a | 0 (0%) | 12 (30%) |  |
| N3b | 0 (0%) | 8 (20%) |  |
| pTNM, n (%) |  |  | **< 0.001** |
| IIA | 16 (40%) | 0 (0%) |  |
| IIB | 1 (2.5%) | 0 (0%) |  |
| IIIA | 3 (7.5%) | 0 (0%) |  |
| IIIB | 0 (0%) | 11 (27.5%) |  |
| IIIC | 0 (0%) | 9 (22.5%) |  |
| Borrmann type, n (%) |  |  | 1.000 |
| Borrmann 2 | 6 (15%) | 6 (15%) |  |
| Borrmann 3 | 13 (32.5%) | 13 (32.5%) |  |
| Borrmann 4 | 1 (2.5%) | 1 (2.5%) |  |
| WHO classification, n (%) |  |  | 0.511 |
| Mucinous | 0 (0%) | 1 (2.5%) |  |
| Poorly differentiated | 6 (15%) | 7 (17.5%) |  |
| Signet ring cell | 4 (10%) | 6 (15%) |  |
| Well to moderately differentiated | 10 (25%) | 6 (15%) |  |
| Lymphatic infiltration, n (%) |  |  | 0.056 |
| Negative | 13 (32.5%) | 6 (15%) |  |
| Positive | 7 (17.5%) | 14 (35%) |  |
| Nerve infiltration, n (%) |  |  | 0.091 |
| Negative | 6 (15%) | 1 (2.5%) |  |
| Positive | 14 (35%) | 19 (47.5%) |  |
| Age, mean ± SD | 56.35 ± 11.46 | 60.4 ± 9.71 | 0.235 |
| CEA, median (IQR) | 2.34 (1.88, 4.25) | 3.38 (1.75, 10.03) | 0.565 |
| CA19-9, median (IQR) | 6.9 (3.9, 18.62) | 13.16 (7.38, 26.64) | 0.233 |
| CA125, median (IQR) | 9.28 (7.6, 12.06) | 7.96 (6.79, 9.58) | 0.117 |

Histological type, T classification, N classification and pTNM classification were according to the AJCC 8th edition of the Cancer Staging Manual of the American Joint Committee on Cancer. Vascular infiltration, nerve infiltration and lymphatic infiltration were determined according to the postoperative pathology report. IQR: Interquartile Range, SD: Standard Deviation.
